# Clinical, laboratory and ultrasonographic findings in 87 cows with type-4 abomasal ulcer

**DOI:** 10.1186/s12917-019-1844-6

**Published:** 2019-03-25

**Authors:** Ueli Braun, Christina Reif, Karl Nuss, Monika Hilbe, Christian Gerspach

**Affiliations:** 1 Department of Farm Animals, Vetsuisse-Faculty, University of Zurich, Winterthurerstrasse 260, CH-8057 Zurich, Switzerland; 2 Institute of Veterinary Pathology, Vetsuisse-Faculty, University of Zurich, Winterthurerstrasse 260, CH-8057 Zurich, Switzerland

**Keywords:** Cattle, Abomasum, Type-4 ulcer, Perforated abomasal ulcer, Peritonitis

## Abstract

**Background:**

This study evaluated the clinical, laboratory, ultrasonographic and pathological findings in 87 cows aged 2 to 10 years (4.5 ± 1.5 years) with type-4 abomasal ulcer.

**Results:**

The most common clinical findings were in decreasing order compromised health status accompanied by partial or complete anorexia (100%), abdominal guarding (81%), congested scleral vessels (77%), ruminal atony (73%), tachycardia (68%), tachypnoea (65%), positive foreign body tests (58%), decreased skin surface temperature (53%), fever (49%), reduction in negative intraabdominal pressure assessed transrectally (39%), poorly subdivided plant fragments in faeces (35%) and arched back (28%). The principal haematological abnormalities were hypokalaemia (72%), haemoconcentration (69%), azotaemia (56%), metabolic acidosis (49%), hyperfibrinogenaemia (45%), leukopenia (35%) and hypoproteinaemia (29%). Other abnormalities were aciduria (56%), haematuria (44%), increased chloride concentration in rumen fluid (34%) and abnormal peritoneal fluid (98%). Of 75 examined cows, 65 (87%) had ultrasonographic evidence of local or generalised peritonitis. On postmortem examination all cows had a type-4 abomasal ulcer and generalised peritonitis. In addition, 36 cows had type-1 ulcers, 6 had type-2 ulcers and one cow had a type-3 ulcer.

**Discussion:**

The clinical signs in cows with type-4 abomasal ulcer are associated with generalised peritonitis. An increased haematocrit, indicating shock-induced haemoconcentration is characteristic in contrast to cows with traumatic reticuloperitonitis. Ultrasonography is useful for visualising and assessing generalised peritonitis.

**Conclusions:**

The diagnosis of type-4 abomasal ulcer based on clinical signs alone is difficult and therefore requires additional diagnostic procedures including the determination of the haematocrit and plasma protein concentration, abdominal ultrasonography and analysis of peritoneal fluid. In most cases, these steps lead to a correct diagnosis and allow timely euthanasia of the cow to prevent further suffering and unnecessary treatment costs.

**Methods:**

The cows underwent a clinical, laboratory, ultrasonographic and postmortem examination.

## Background

Abomasal ulcer disease is of great importance in cattle. Abomasal lesions are divided into erosions and ulcers [1]; erosions are superficial lesions of the mucous membrane and ulcers are deep defects that penetrate the basement membrane of the abomasal mucosa [2]. Abomasal ulcers vary in number and size and can heal spontaneously, but this is associated with the formation of a permanent scar [1]. Abomasal ulcers were previously divided into four types [1, 3], but a new system that has been used for a number of years classifies ulcers into five types [4]. Classification is based on the depth of penetration, the degree of haemorrhage and the degree of peritonitis caused by the ulcer. Type 1 denotes erosions and/or non-perforated ulcers with minimal haemorrhage; type 2 is associated with severe intraluminal haemorrhage because of erosion of a large blood vessel; type 3 is a perforated ulcer located close to neighbouring organs or the peritoneum resulting in acute local peritonitis and adhesions and sometimes the formation of abscesses; and type 4 is a perforated ulcer accompanied by diffuse peritonitis because of contamination of the abdominal cavity with ingesta from the abomasum. Abomasal perforation into the omental bursa associated with localised omental bursitis was previously considered a subset of type-3 ulcer [5] but has since been designated type 5 [4]. Individual cows may have multiple ulcers from more than one classification [2].

The clinical signs vary widely depending on the type of abomasal ulcer; generalised peritonitis is common in cows with type-4 ulcer and is often fatal within 24 to 48 h [3]. The clinical signs in cows with type-4 ulcer resemble those of septic shock and include tachycardia, tachypnoea, fever, congested scleral vessels, pale and muddy mucous membranes, decreased skin surface temperature, spontaneous grunting and abdominal guarding [6, 7]. Abdominal pain was observed in five of seven (71%) [1] and ten of 22 cows (45%) with a perforated ulcer [6], respectively. Transrectal palpation reveals absence of or a reduction in the normally negative intraabdominal pressure and the serosal surfaces may have a sandpaper-like texture. Almost all cows have diarrhoea [6, 7]. Increased haematocrit (> 35%) accompanied by a decrease in total protein (< 60 g/l) and leukocytosis attributable to neutrophilia, often with a left shift, are common [6, 7]. Metabolic acidosis has also been reported in cows with type-4 abomasal ulcer [7].

Ultrasound examination can be helpful in assessing the position, size, wall and content of the abomasum and possible inflammatory lesions that involve neighbouring organs [8]. However, mucosal erosions and type-1 ulcers that were diagnosed at postmortem in 16 of 50 (32%) clinically healthy cows were missed ultrasonographically [9]. When blood is aspirated during abomasocentesis, the likelihood of abomasal ulcer is high [10]. Ultrasonography was identified as the most important diagnostic procedure in a cow with abomasal lymphosarcoma [11]; a thickened abomasal wall and prominent abomasal leaves accompanied by enlarged lymph nodes were considered diagnostic. Ultrasonographic findings in cows with peritonitis vary widely [8]. Fibrinous peritonitis is characterised by echogenic deposits on the peritoneum and organ surfaces in the absence of effusion, but fibrin may have fluid inclusions. Anechoic to hypoechoic fluid accumulation (inflammatory ascites) interspersed with fibrin strands between organs may be seen in generalised peritonitis. With a perforated ulcer, ingesta can be seen as an echogenic coating on various abdominal organs.

Because the clinical diagnosis of type-4 abomasal ulcer is not straightforward, and detailed investigations of large numbers of cows with this ulcer type are lacking, the purpose of this study was to describe the clinical, laboratory and ultrasonographic findings of cows with type-4 abomasal ulcer to facilitate the diagnosis of this disease.

## Methods

### Animals

**Fig. 1:**
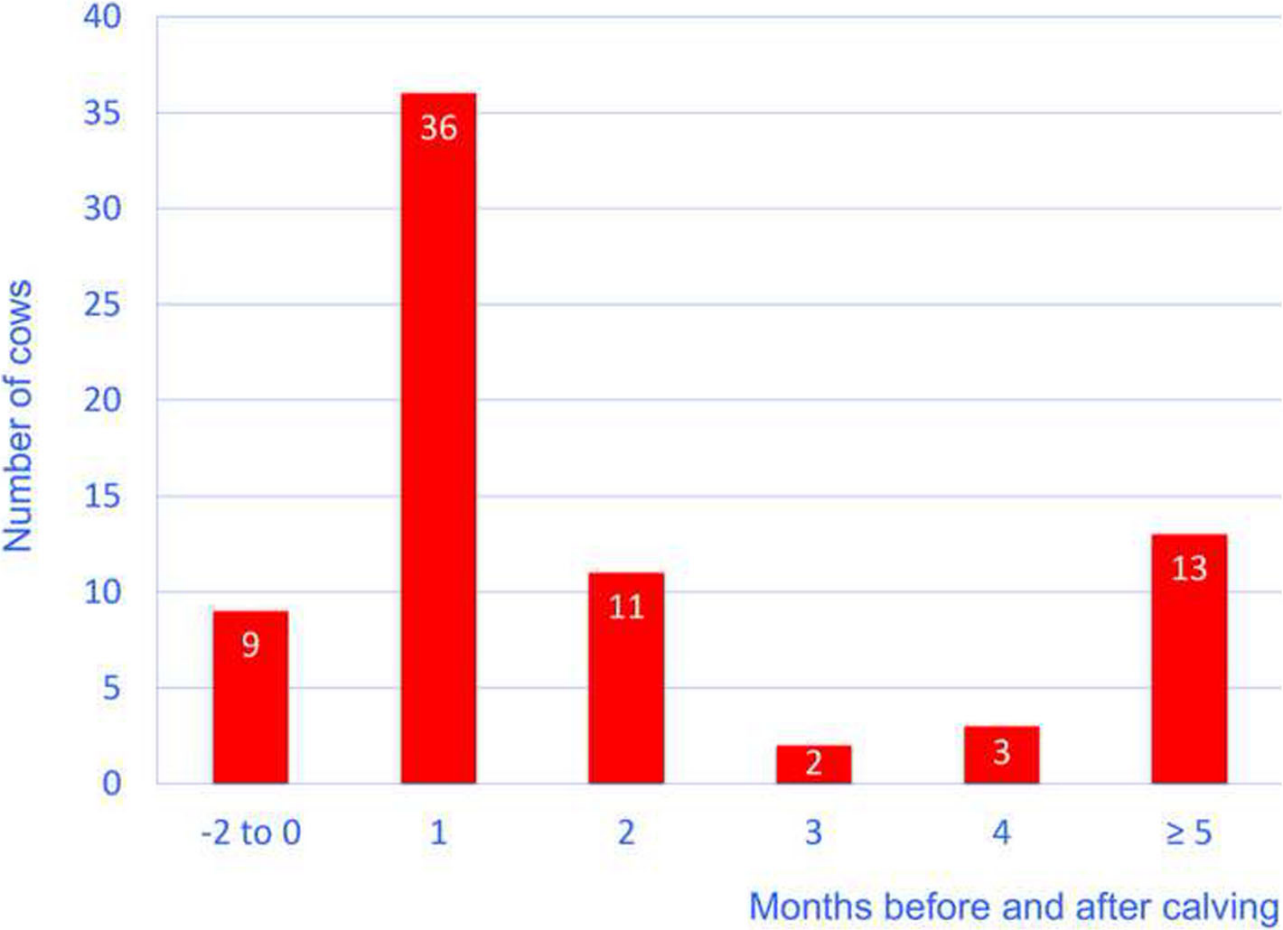
Frequency of type-4 abomasal ulcers in 87 cows during the lactation cycle

This was a retrospective study of 87 cows that had a main diagnosis of type-4 abomasal ulcer. The cows had been admitted to the Veterinary Teaching Hospital, University of Zurich, from January 1, 1991 to December 31, 2014. The final diagnosis was based on the results of postmortem examination. The results were described in detail [12]. The cows ranged in age from 2 to 10 years (mean ± standard deviation [sd], 4.5 ± 1.5 years). Breeds included Brown Swiss (36), Holstein-Friesian (25), Swiss Fleckvieh (24) and crossbred cattle (2). The duration of illness was < 2 days in 36 cows, 2 to 6 days in 40 cows, 7 to 14 days in 6 cows and > 14 days in 4 cows. The majority of cows (*n* = 36, 49%) became ill within 4 weeks after calving (Fig. 1); this incidence was significantly higher than that of other stages of lactation (*P* < 0.01). Twenty-four cows had been treated with non-steroidal anti-inflammatory drugs (NSAIDs), six with corticosteroids and another six with NSAIDs and corticosteroids before referral, but the exact dosages were not known.

### Clinical examination

The cows underwent a thorough clinical examination [13]. General health was evaluated by determining demeanour, appearance of hair coat and muzzle, skin elasticity, position of the eyes in the sockets and skin surface temperature. Each cow was observed for signs of pain such as spontaneous grunting and bruxism. The rumen was assessed for degree of fill, number and intensity of contractions and content stratification. Sensitivity in the reticular region was assessed by preventing the animal from breathing for a short period by placing a plastic rectal sleeve over the mouth and nose and listening for grunting during the ensuing deep breath. This was followed by foreign body tests, which included the pole test, back grip and percussion of the abdominal wall over the region of the reticulum with a rubber hammer. Each test was carried out 4 times as described [14], and the reaction of the animal was observed each time. A test was considered positive when it elicited a short grunt at least three out of four times. The response to a test was considered questionable when it elicited a grunt two out of four times and negative when the animal did not grunt or grunted only once. Ballottement and simultaneous auscultation as well as percussion and simultaneous auscultation of the abdomen on both sides and rectal examination were also carried out. Faeces were assessed for colour, consistency, amount, fibre particle length and abnormal contents.

### Laboratory analyses

The following blood samples were collected from all cows: 5 ml of EDTA blood for haematological analysis, 10 ml of whole blood for serum biochemistry, 2 ml of whole blood mixed with 0.2 ml heparin for venous blood gas analysis and 5 ml of EDTA blood for the glutaraldehyde test. Haematological analysis included the determination of haematocrit, total leukocyte count and the concentrations of fibrinogen and total protein using an automated blood analyser (CELL-Dyn 3500, Abbott Diagnostics Division, Baar). The concentration of serum urea nitrogen was determined at 37 °C using an automated analyser (Cobas-Integra-800-Analyser, Roche Diagnostics, Basel) and the manufacturer’s reagents (Roche Reagents) according to the International Federation of Clinical Chemistry and Laboratory Medicine (IFCC). Venous blood gas analysis was done using an automated analyser (RapidLab 248, Siemens Schweiz AG, Zurich). A glutaraldehyde test (Glutaltest®, Graeub AG, Bern) was done according to the manufacturer’s instructions. Results were interpreted relative to reference intervals recently reported [15].

A urine sample, mainly collected during spontaneous micturition, was analysed in 76 cows. The colour and transparency of the urine were assessed macroscopically, and the specific gravity was determined using a refractometer (HRMT 18, A. Krüss Optronic GmbH, Hamburg, Germany). A urine test strip (Combur9®, Roche, Basel) was used to determine pH and the presence of protein, erythrocytes, glucose, ketones, leukocytes, nitrite, urobilinogen and bilirubin.

A sample of rumen fluid (200 to 300 ml) was collected using a Dirksen probe [13] in 67 cows and assessed for colour, odour, consistency and pH. In addition, the methylene blue reduction time and the concentration of chloride were determined. The concentration of chloride in rumen fluid was carried out using an MK-II-Chloride Analyser 9265 (Sherwood, Cambridge).

### Ultrasonographic examination and abdominocentesis

Seventy-five cows underwent ultrasonographic examination of the reticulum (*n* = 58), abomasum (*n* = 21) and abdomen (*n* = 63) using a 3.5 or 5.0 MHz convex or linear transducer [16]. Echogenic deposits, with or without hypoechoic or anechoic fluid inclusions, and structures of various shapes and echogenicities with central echogenic fluid collections, reflect inflammatory changes of the peritoneum that include fibrinous deposits and abscesses were interpreted and referred to as inflammatory lesions of the peritoneum [16, 17].

Amount and appearance of fluid accumulations and fibrin strands were noted. Forty-seven cows with ultrasonographic evidence of abdominal fluid accumulation underwent ultrasound-guided abdominocentesis [18] and the aspirated fluid was assessed macroscopically with respect to colour, odour and transparency. Specific gravity and total solids were measured with a refractometer. The aspirated fluid was considered an exudate when at least one of the following criteria was met: specific gravity > 1.015, total solids > 30 g/l, cloudy appearance, malodourous appearance and green discolouration.

### Euthanasia

All severely affected cows that did not die spontaneously were euthanased immediately after the initial examination or after 2 to 4 days of unsuccessful treatment. Euthanasia was done with pentobarbital (Esconarkon, Streuli Pharma), 80 mg/kg body weight intravenously.

### Statistical analysis

The program IBM SPSS Statistics 22.0 was used for analysis. Frequencies were determined for each variable. The Wilk-Shapiro test was used to test the data for normality. Means ± standard deviations were calculated for normal data (total protein) and medians for non-normal data (heart rate, respiratory rate, rectal temperature, haematocrit, white blood cell count, fibrinogen and urea concentration, glutaraldehyde test coagulation time, pH, pCO_2_, HCO_3_^−^ and base excess of venous blood, urine pH, urine specific gravity). Differences in ulcer occurrence at various stages of lactation were analysed using a one-way analysis of variance and the post hoc Bonferroni test. A value of *P* < 0.05 was considered significant.

## Results

### Overview of the most common clinical findings

**Fig. 2:**
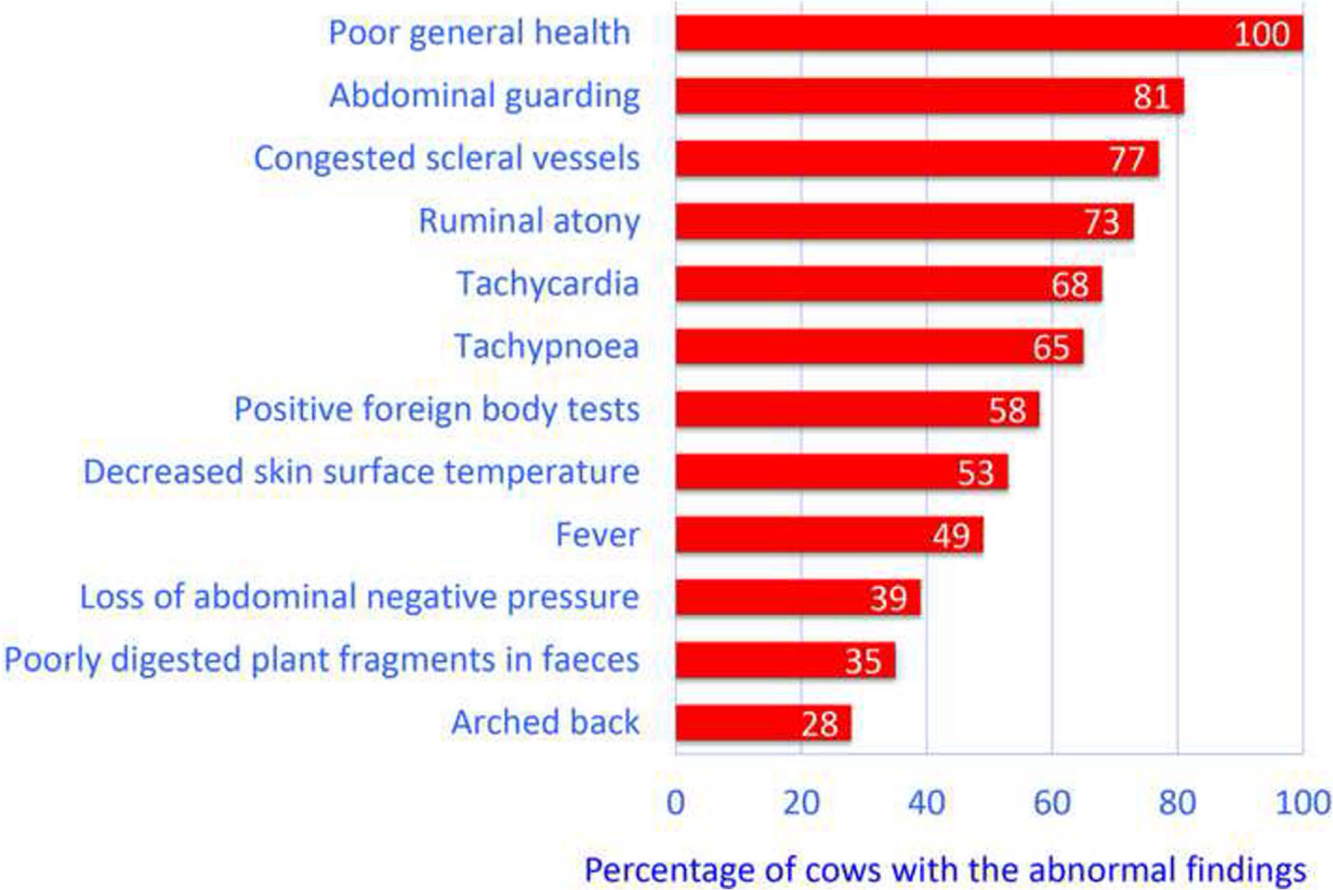
Most common abnormal clinical findings in 87 cows with type-4 abomasal ulcer

The most common clinical findings in decreasing frequency were compromised health status accompanied by partial or complete anorexia (100%), abdominal guarding (81%), congested scleral vessels (77%), ruminal atony (73%), tachycardia (68%), tachypnoea (65%), positive foreign body tests (58%), decreased skin surface temperature (53%), fever (49%), reduction in negative intraabdominal pressure assessed transrectally (39%), poorly digested plant fragments in faeces (35%) and arched back (28%) (Fig. 2).

### Health status, behaviour, posture and signs of pain

All cows were ill and had reduced appetite or anorexia. Five (6%) cows were down at the time of admission, 24 (28%) were obtunded, 13 (15%) had muscle tremors and 24 (28%) had an arched back. Six (7%) cows had droopy ears and 4 (5%) had a droopy head. Two (2%) cows had a sawhorse stance and two others had abducted elbows. The principal signs of abdominal pain were bruxism (*n* = 22, 25%), spontaneous grunting (*n* = 16, 18%) and colic (restlessness and weight shifting in fore- and hind limbs, sunken back, kicking at the belly; *n* = 7, 8%).

### Circulatory and respiratory systems and temperature

The heart rate ranged from 44 to 196 beats/min (median, 98 beats/min) (Table 1) and 59 (68%) cows had tachycardia. The respiratory rate ranged from 14 to 80 breaths/min (median, 32 breaths/min) and was increased in 56 (65%) cows. The rectal temperature ranged from 36.0 to 40.5 °C (median, 38.9 °C); 24 (28%) cows had hypothermia and 43 (49%) had pyrexia. The skin surface temperature was lower than normal in 45 (53%) cows and warmer than normal in four (5%). Sixty-six (77%) cows had congested scleral vessels, 23 (27%) had pale mucous membranes and six (7%) cows each had hyperaemic and cyanotic mucous membranes.

**Table 1:** Clinical findings in cows with type-4 abomasal ulcer

Variable	Finding	Number of cattle	%
Heart rate (n = 87, median = 98 bpm)	Normal (60–80)	26	30
	Decreased (44–59)	2	2
	Mildly increased (81–100)	23	26
	Moderately increased (101–120)	23	26
	Severely increased (121–196)	13	15
Respiratory rate	Normal (15–25)	28	33
(*n* = 86, median = 32 breaths per min.)	Decreased (14)	2	2
	Mildly increased (26–35)	23	27
	Moderately increased (36–45)	16	18
	Severely increased (46–80)	17	20
Rectal temperature (*n* = 87, median = 38.9 °C)	Normal (38.4–38.9)	20	23
	Decreased (36.0–38.3)	24	28
	Mildly increased (39.0–39.4)	17	20
	Moderately increased (39.5–40.0)	23	26
	Severely increased (40.1–40.5)	3	3
Rumen motility (*n* = 86)	Normal	1	1
	Decreased	22	26
	Absent	63	73
Foreign body tests (*n* = 76)	All negative	32	42
	Pole test positive^1^	35	46
	Back grip positive^1^	31	41
	Percussion of the reticular region positive^1^	25	33
	1 of 3 tests positive	12	16
	2 of 3 tests positive	18	24
	3 of 3 tests (all tests) positive	14	18
Swinging and per-cussion auscultation on the left side (n = 87)	Both negative (normal)	74	85
	Only swinging auscultation positive	3	3
	Only percussion auscultation positive	4	5
	Both tests positive	6	7
Swinging and per-cussion auscultation on the right side (*n* = 87)	Both negative (normal)	43	49
	Only swinging auscultation positive	17	20
	Only percussion auscultation positive	9	10
	Both tests positive	18	21
Rectal findings (*n* = 87)	Normal findings	44	51
	Loss of negative pressure	34	39
	Sandpaper-like texture of serosa	9	10
	Rumen dilated	14	16
	Gaseous distension of the small intestines	5	6
	Miscellaneous abnormal findings	8	9
	Faecal output reduced	57	66
	Empty rectum	12	14

^1^Positive: at least 3 of 4 attempts elicited a grunt

### Digestive system

Sixty-eight (81%) cows had abdominal guarding and six (7%) had abdominal distension. Rumen motility was reduced in 22 (26%) cows and absent in 63 (73%) (Table 1). Of the 76 cows that underwent foreign body testing, 35 (46%) reacted to the pole test, 31 (41%) to the back grip and 25 (33%) to percussion of the reticular area. Twelve (16%) cows had one positive test, 18 (24%) cows had two and in 14 (18%), all three tests were positive. In 32 (42%) cows, all three tests were negative or equivocal and 44 (58%) cows had at least one positive test. Swinging and/or percussion and auscultation was positive in 13 (15%) cows on the left and in 44 (51%) on the right. Fifty-five (63%) cows had abnormal transrectal palpation findings; 34 (39%) had partial or complete loss of negative abdominal pressure and 9 (10%) cows had serosal surfaces with a sandpaper-like texture. The rumen was dilated in 14 cows and gaseous distension of the small intestines was diagnosed in five cows. Eight cows had miscellaneous abnormal rectal findings including caecal dilatation and right displacement of the abomasum characterised by a gas-filled area of the abomasum. Faecal output was reduced in 57 (66%) cows and 12 (14%) cows had an empty rectum. Faecal consistency varied widely from loose to firm and only 14 (19%) cows had diarrhoea and passed thin semifluid to watery faeces. The colour of the faeces was dark to black in 12 (16%) cows and the degree of comminution was moderate to poor in 26 (35%) cows.

### Laboratory findings (blood, urine, rumen fluid)

**Fig. 3:**
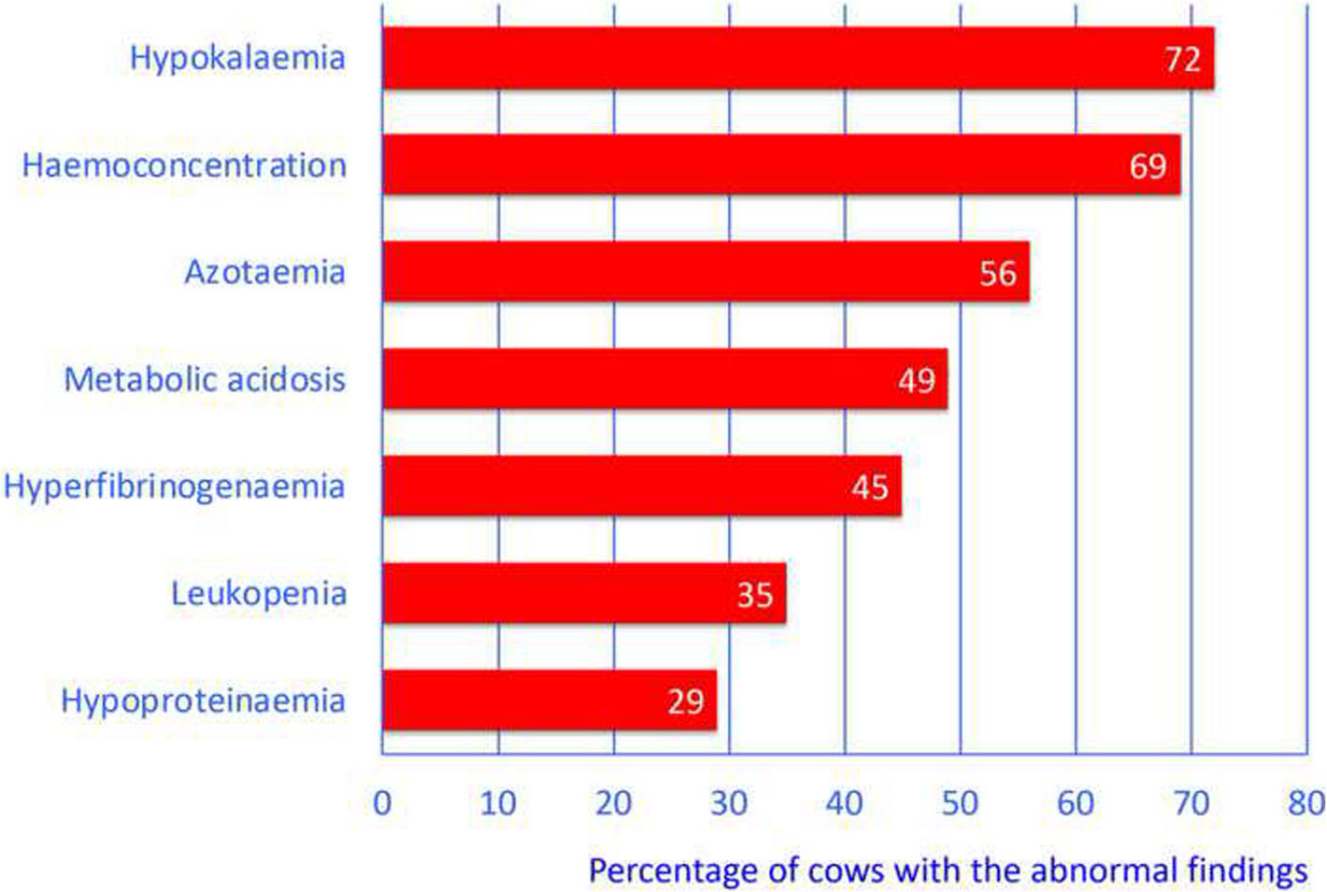
Most common abnormal blood variables in 87 cows with type-4 abomasal ulcer

The most important abnormal blood variables in decreasing frequency were hypokalaemia (72%), haemoconcentration (69%), azotaemia (56%), metabolic acidosis (49%), hyperfibrinogenaemia (45%), leukopenia (35%) and hypoproteinaemia (29%) (Fig. 3). The haematocrit was decreased (11 to 29%) in 11 (13%) cows and increased (36 to 62%) in 60 (69%) cows (Table 2). Leukocyte numbers ranged from 1600 to 24,600/μl blood; 30 (35%) cows had leukopenia (< 5000 leukocytes/μl blood) and 16 (19%) cows had leukocytosis (> 10,000 leukocytes/μl blood). Total protein concentration was decreased (40 to 59 g/l) in 25 (29%) cows and increased (81 to 93 g/l) in 10 (11%) cows). Seventeen (20%) cows had hypoproteinaemia (< 60 g total protein/l) combined with haemoconcentration (haematocrit > 35%). Hyperfibrinogenaemia (8 to 13 g/l) occurred in 39 (45%) cows. Forty-eight (56%) cows had azotaemia (blood urea nitrogen > 6.6 mmol/l). The glutaraldehyde coagulation test time was shortened (< 10 min) in 17 (22%) cows and prolonged (16 to 60 min) in 7 (9%) cows. Venous blood gas analysis showed a decreased pH (< 7.4) in 35 (49%) cows and an increased pH in 18 (25%) cows.

**Table 2:** Haematological and blood biochemical findings in cows with type-4 abomasal ulcer

Variable (mean ± sd or median)	Finding	Number of cattle	Percent
Haematocrit (%) (*n* = 87; median, 40%)	Normal (30–35)	16	18
	Decreased (11–29)	11	13
	Increased (36–62)	60	69
White blood cell count (/μl) (*n* = 86; median, 6300/μl)	Normal (5000 – 10,000)	40	46
	Decreased (1600 – 4999)	30	35
	Increased (10,001 – 24,600)	16	19
Total protein concentration (*n* = 87; mean ± sd, 65.3 ± 12.5 g/l)	Normal (60–80)	52	60
	Decreased (40–59)	25	29
	Increased (81–93)	10	11
Fibrinogen concentration (*n* = 86; median, 6.0 g/l)	Normal (4–7)	39	45
	Decreased (2–3)	8	10
	Increased (8–13)	39	45
Urea concentration (*n* = 86; median, 7.3 mmol/l)	Normal (2.4–6.5)	38	44
	Increased (6.6–28.6)	48	56
Potassium concentration (*n* = 86; median, 3.6 mmol/l)	Normal (4.0–5.0)	17	20
	Decreased (1.6–3.9)	62	72
	Increased (5.1–7.5)	7	8
Glutaraldehyde test (*n* = 76; median, 10.0 min.)	Normal (10–15)	52	69
	Decreased (1.5–9.9)	17	22
	Prolonged (1.5–9.9)	7	9

**Fig. 4:**
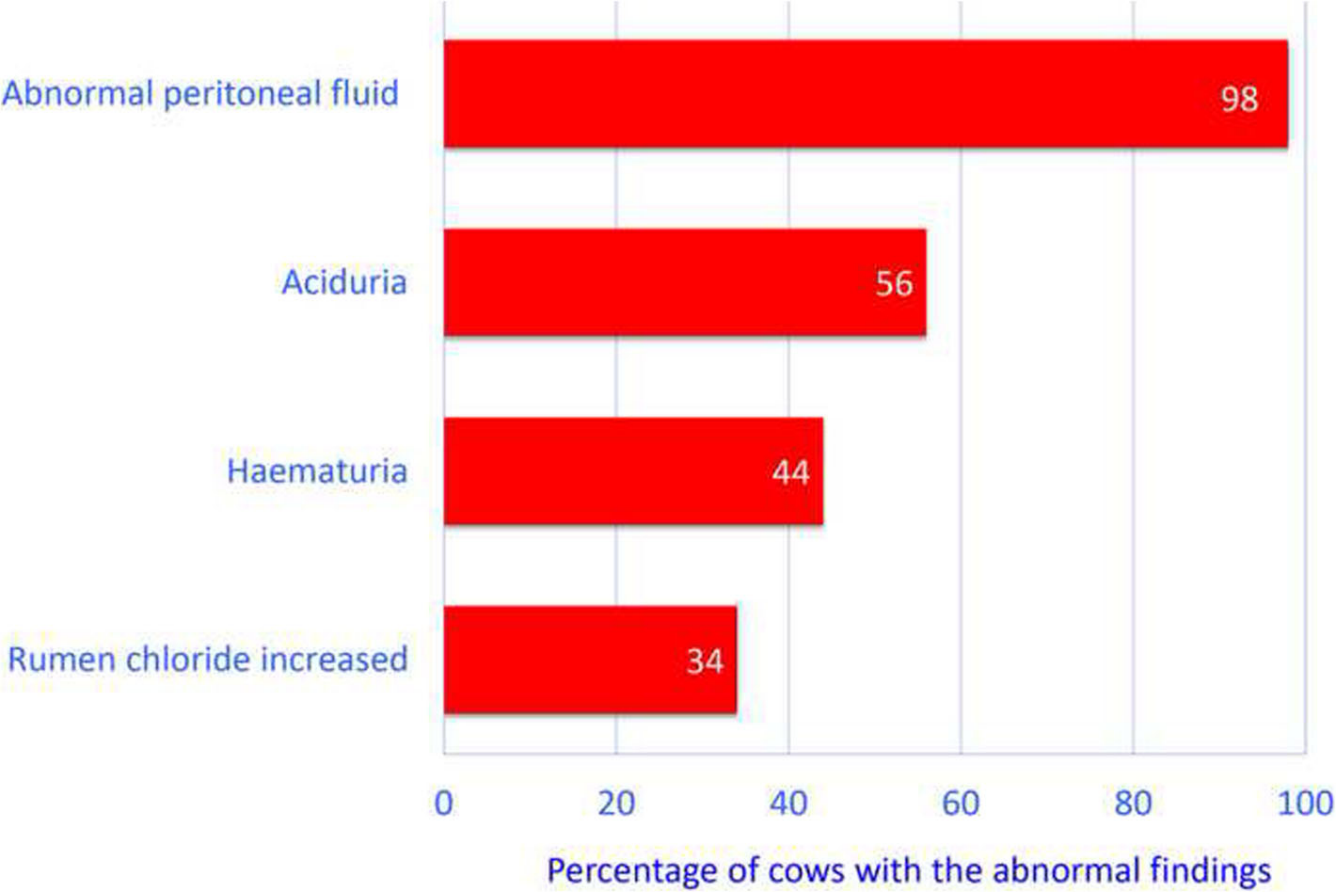
Most common abnormal findings in peritoneal fluid, urine and rumen fluid from 87 cows with type-4 abomasal ulcer

Urinalysis showed aciduria (pH < 6.5) in 35 (56%) cows and haematuria (5 to 250 erythrocytes per high-power field) in 33 (44%) cows with macroscopically normal urine (Fig. 4). Six (8%) cows had proteinuria (1 to 1.5 g/l), 8 (11%) had ketonuria (0.1 to 1.5 g/l) and 18 (24%) had glucosuria (0.5 to 10.0 g/l) (not shown in Fig. 4). The chloride concentration in rumen fluid was increased (> 25 mmol/l) in 23 (34%) cows. The sample of aspirated peritoneal fluid was an exudate in 46 (98%) of 47 cows and normal in the remaining cow; 39 samples were yellow, 5 were haemorrhagic and the remaining 2 were green. Twenty-two samples were opaque and 3 were malodorous. The specific gravity varied from 1.013 to 1.045 (mean ± sd, 1.029 ± 8.6) and the protein concentration from 4 to 76 g/l (median, 36.4 g/l).

### Ultrasonographic findings

**Fig. 5:**
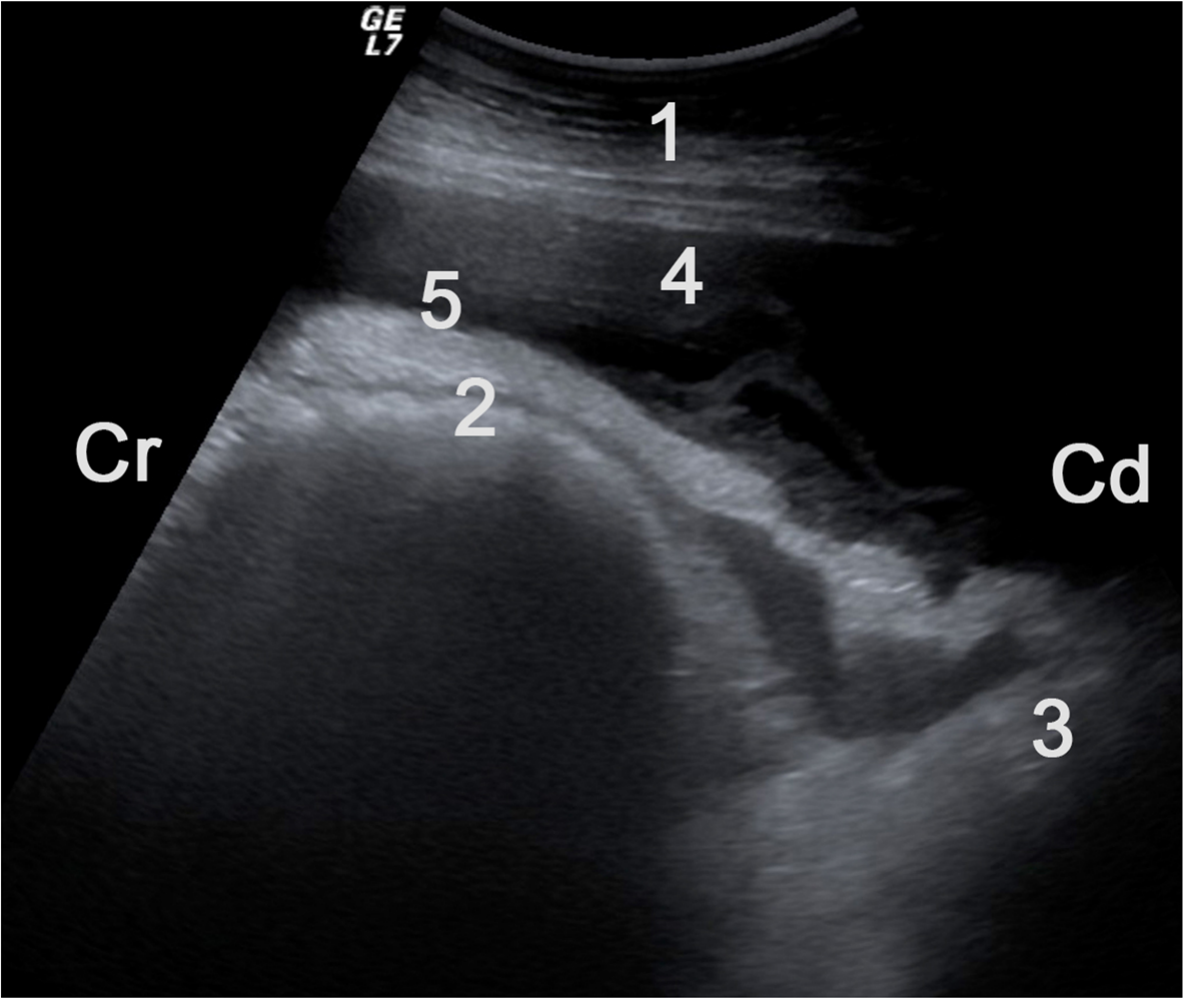
Ultrasonogram of the reticulum obtained from the ventral abdominal wall showing fibrinous changes and effusion in the reticular area in a 2.6-year-old Holstein-Friesian cow with type-4 abomasal ulcer. A 5.0-MHz convex transducer was used. 1 Ventral abdominal wall, 2 Reticulum, 3 Anterior dorsal blind sac of rumen, 4 Extensive fluid accumulation ventral and caudal to reticulum, 5 Fibrin on the serosal surface of the reticulum extending to dorsal blind sac of rumen, Cr Cranial, Cd Caudal

**Fig. 6:**
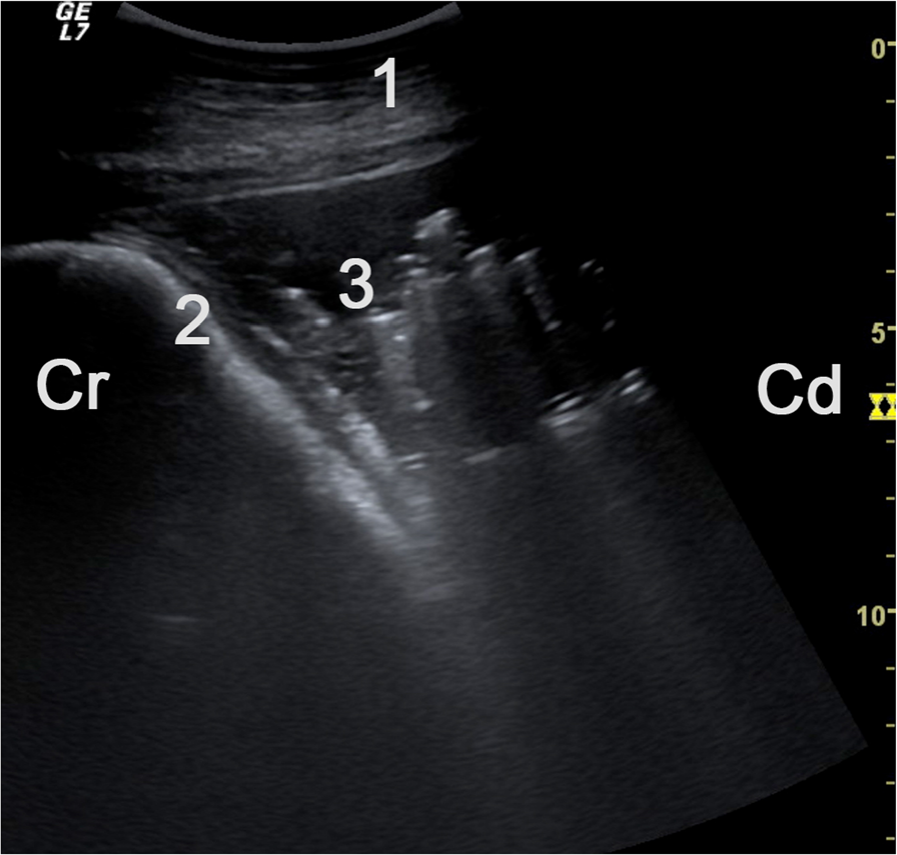
Ultrasonogram of the reticular area obtained from the ventral abdominal wall showing fluid accumulation with gaseous inclusions in a 3.9-year-old Brown-Swiss cow with type-4 abomasal ulcer. A 5.0-MHz linear transducer was used. The echogenic stippling in the fluid represents gas produced by bacteria. The aspirated peritoneal fluid was opaque and the specific gravity and protein concentration were increased at 1.033 and 50 g/l, respectively. The fluid contained 225 cells/μl and cytological examination of a smear showed many bacteria (which were responsible for gas production). 1 Ventral abdominal wall, 2 Reticulum, 3 Fluid with gaseous inclusions, Cr Cranial, Cd Caudal

**Fig. 7:**
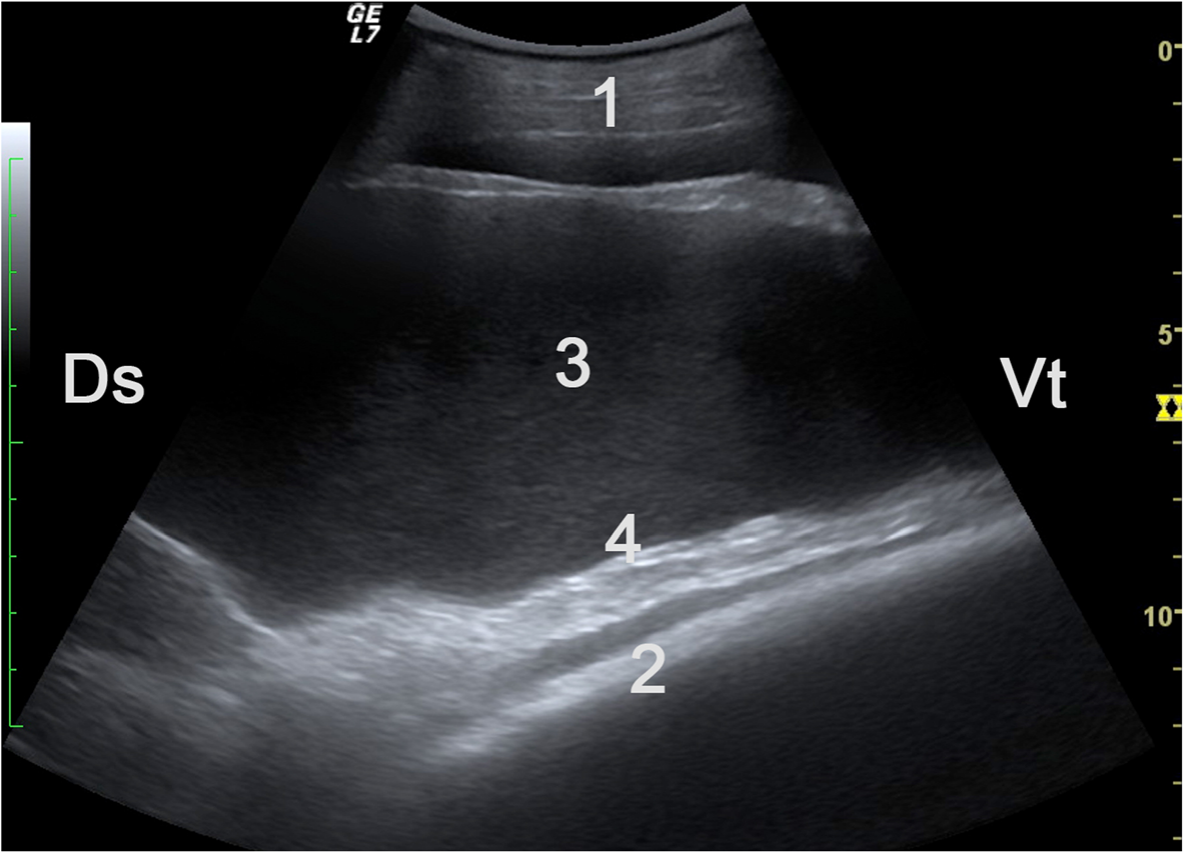
Ultrasonogram obtained from the left flank showing massive hypoechoic effusion in a 2-year-old Holstein-Friesian cow with type-4 abomasal ulcer and left displacement of the abomasum. A 5.0-MHz convex transducer was used. 1 Abdominal wall in the left flank, 2 Rumen, 3 Massive accumulation of hypoechoic fluid between rumen and left abdominal wall, 4 Thickened greater omentum, Ds Dorsal, Vt Ventral

**Fig. 8:**
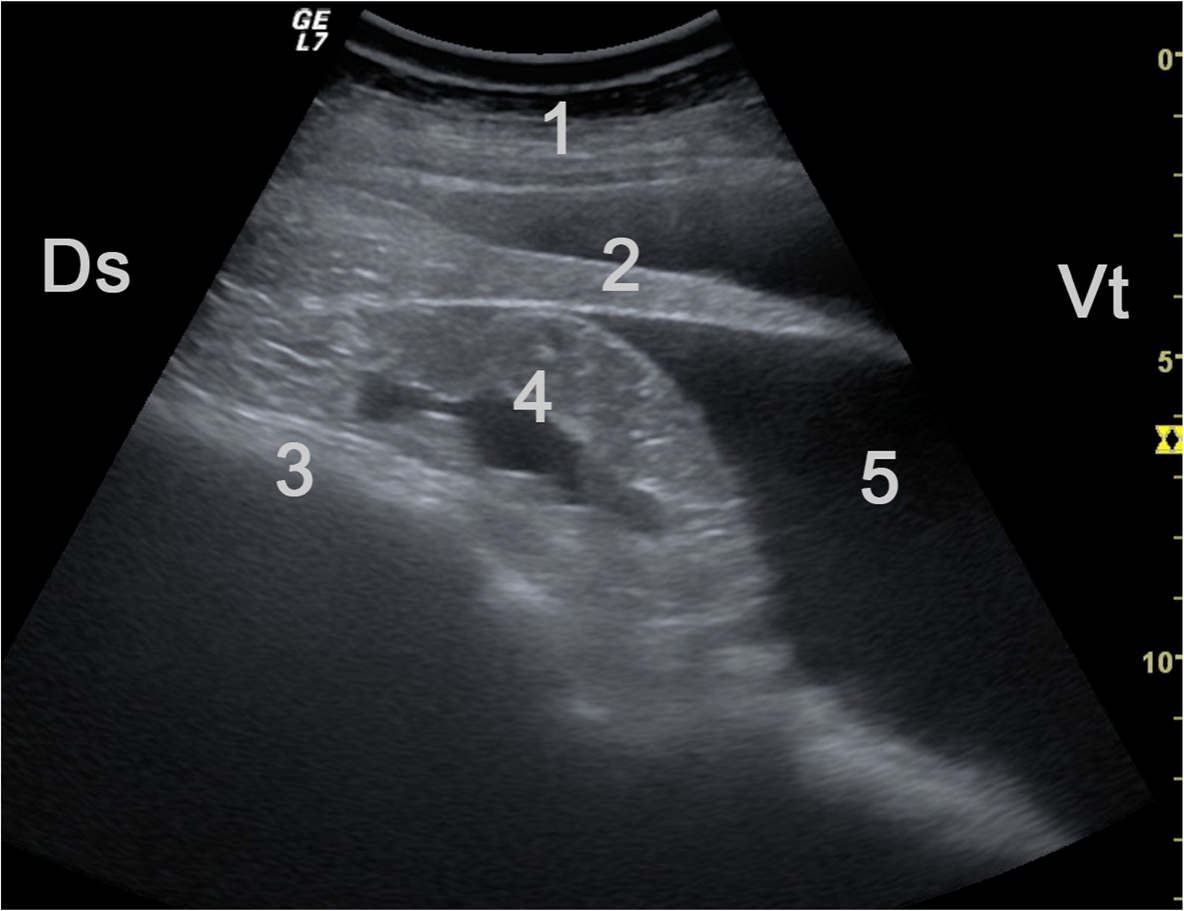
Ultrasonogram obtained from the left 7th intercostal space of the cow in Fig. 7 showing extensive intraabdominal inflammatory lesions. A 5.0-MHz convex transducer was used. The spleen is surrounded by hypoechoic fluid, and echoic inflammatory changes interspersed with fluid are seen between the spleen and rumen. 1 Lateral abdominal wall, 2 Spleen, 3 Rumen, 4 Echoic inflammatory changes interspersed with fluid, 5 Fluid accumulation, Ds Dorsal, Vt Ventral

**Fig. 9:**
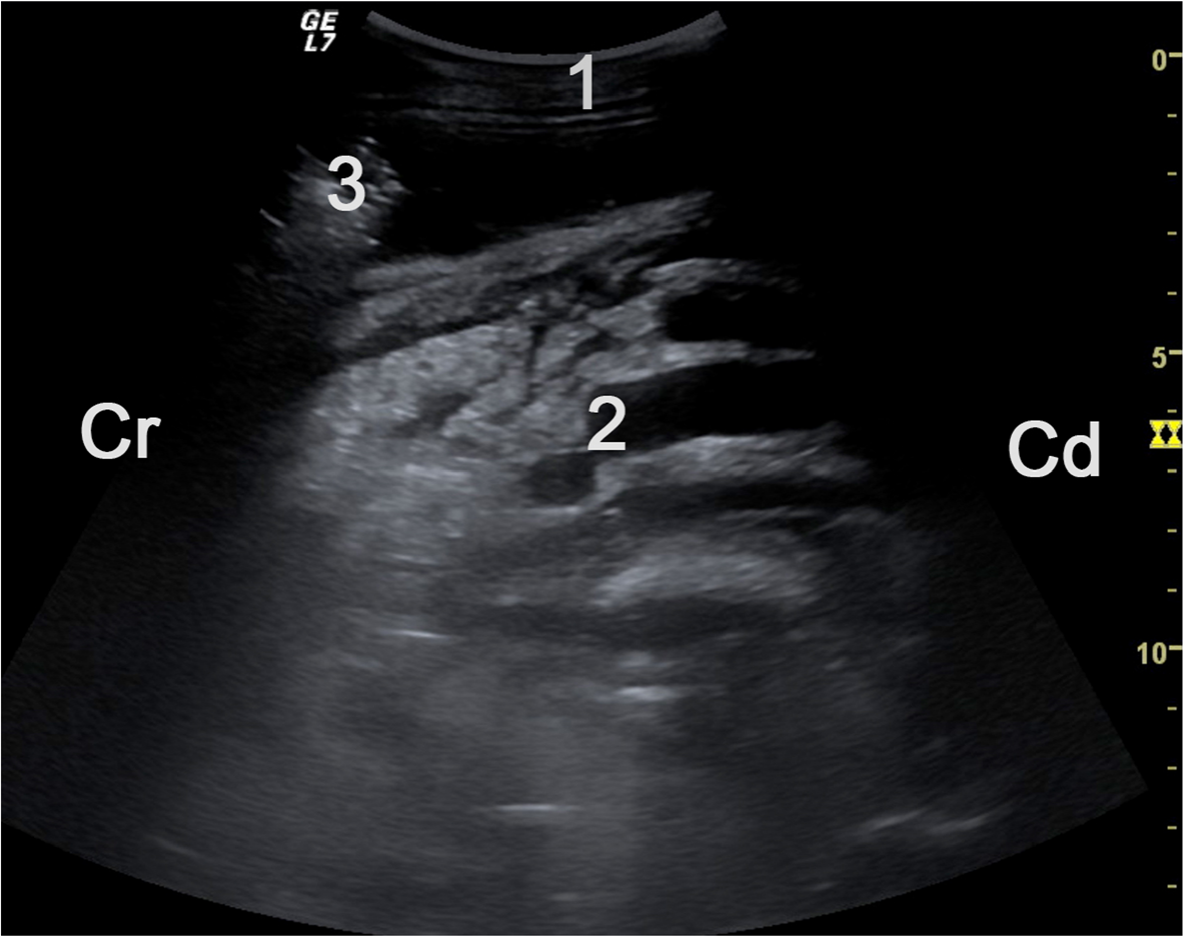
Ultrasonogram obtained from the right paramedian area caudal to the reticulum of the cow of Fig. 6 showing extensive abdominal fibrinous lesions. A 5.0-MHz convex transducer was used. Massive echoic changes interspersed with fluid representing fibrinous peritonitis are seen between the abdominal wall and the abomasum (not visible). 1 Ventral abdominal wall, 2 Echoic lesions interspersed with fluid, 3 Fluid with gaseous inclusions (similar to those shown in Fig. 6). Cr Cranial, Cd Caudal

The reticulum was raised from the ventral abdominal wall in 20 cows, had an abnormal contour in 19 cows and decreased amplitude of contraction in 12 cows (Table 3). Reticular atony was diagnosed in 36 cows, echogenic changes with or without fluid inclusions in 40 cows and free fluid in the reticular area in seven cows (Figs. 5 and 6). Other findings were abomasal dilatation in five cows, left displacement of the abomasum in three and right displacement of the abomasum in four cows. Fourteen cows had fibrinous changes and/or free abdominal fluid associated with the abomasum. When an ulcer was accompanied by left displacement of the abomasum, massive inflammatory changes were also seen adjacent to the left abdominal wall (Figs. 7 and 8). Generalised echogenic changes (Fig. 9) were seen in 36 cows and generalised fluid accumulations in seven cows. Overall, 65 (87%) of the 75 scanned cows had ultrasonographic evidence of local or generalised peritonitis.

**Table 3:** Ultrasonographic findings in cows with type-4 abomasal ulcer

Location	Findings	Number of cows
Reticulum (*n* = 58)	Elevated from ventral abdominal wall	20
	Contour abnormal	19
	Amplitudes of contraction decreased	12
	Reticular atony	36
	Echogenic changes with or without fluid inclusions	40
	Free fluid in reticular region	7
Abomasum (*n* = 47)	Dilated	5
	Left displacement	3
	Right displacement	4
	Fibrin deposits on serosa	7
	Free fluid in abomasal region	7
Abdomen	Generalised echogenic lesions	36
	Generalised free fluid	7

### Concurrent diseases

Comorbidity occurred in 29 (33%) cows; 20 cows had one, seven cows had two and two cows had three or more additional diseases. The most common problems were claw disorders (*n* = 24), left or right displacement of the abomasum (*n* = 7), fatty liver syndrome (*n* = 6), metritis or endometritis (*n* = 6), fascioliasis (*n* = 6), mastitis (*n* = 1) and ketosis (*n* = 1).

### Diagnosis and treatment attempts

A tentative diagnosis of type-4 abomasal ulcer was made in 66 (76%) cows, and in 21 (24%) cows, the diagnosis was not clear. Fifty-four (62%) cows in which a type-4 ulcer was diagnosed were euthanased immediately except for a few that died during the examination. Twelve (14%) cows underwent right flank exploratory laparotomy to confirm the diagnosis and all were euthanased because of generalised peritonitis. The cows with an unclear diagnosis received a continuous intravenous infusion of a sodium-chloride-glucose solution (10 l per day, 9 g sodium chloride and 50 g glucose per litre), antibiotics (procaine penicillin G procaine, 12,000 IU/kg body weight) and an NSAID (flunixin meglumine, 1 mg/kg), and two cows also received a magnet. Six cows died after the start of treatment and the remaining 15 were euthanased after two to four days because of deterioration in condition.

### Postmortem diagnosis

**Fig. 10:**
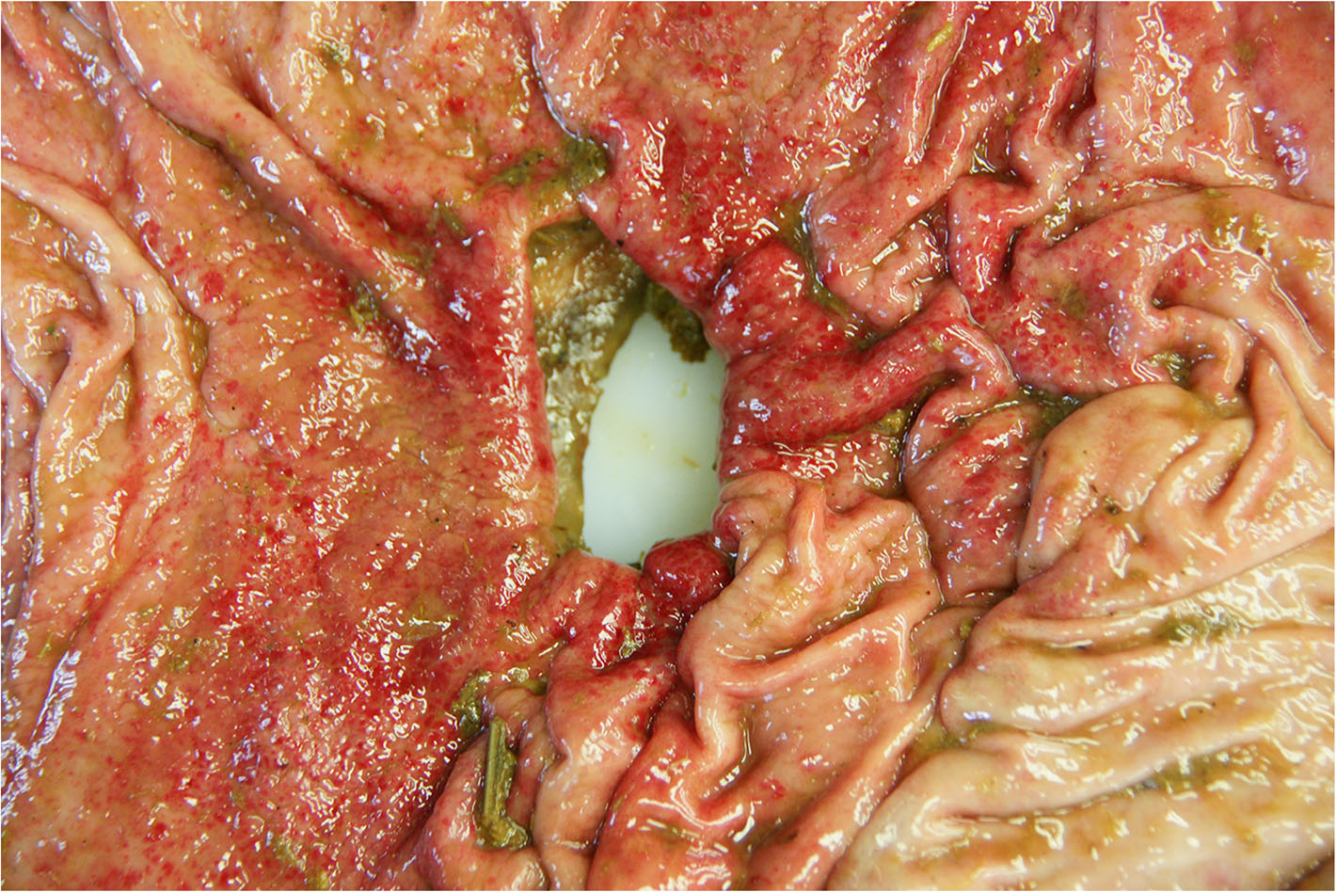
Postmortem view of the luminal aspect of the abomasum with a type-4 ulcer in a 5-year-old Fleckvieh cow

**Fig. 11:**
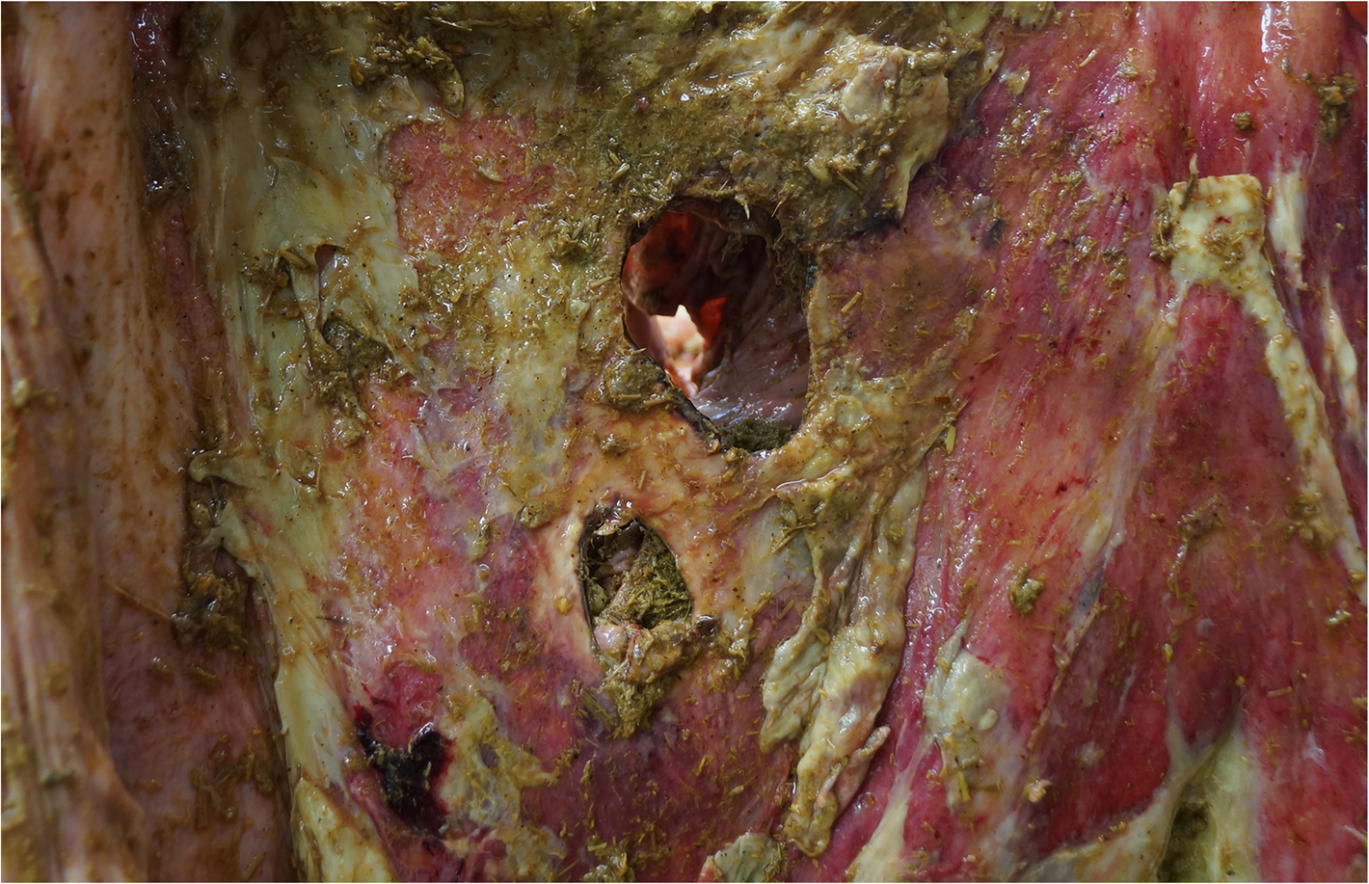
Postmortem view of the serosal aspect of the abomasum with type-4 ulcers in a 7.5-year-old Fleckvieh cow. There are two perforated ulcers and the serosa is covered with ingesta and fibrin

**Fig. 12:**
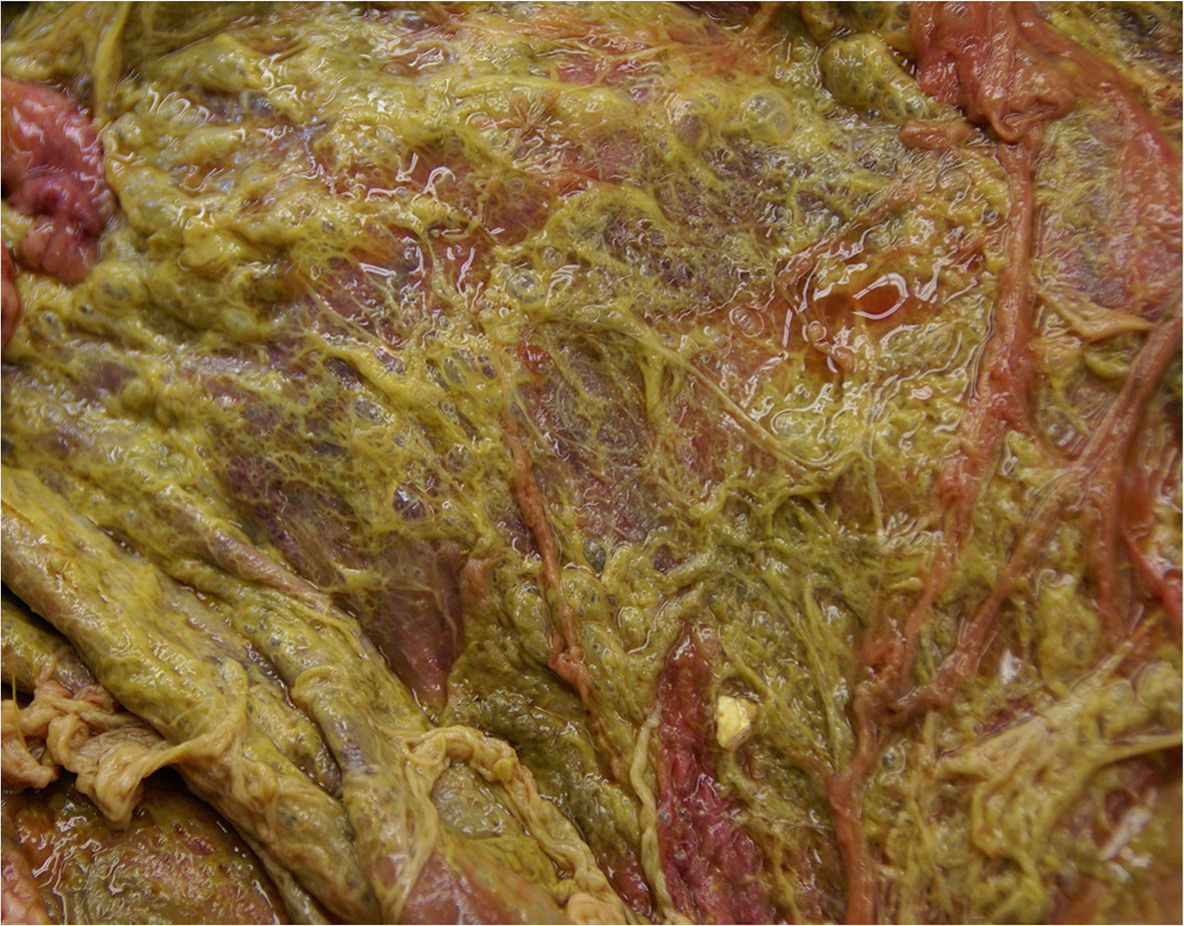
Postmortem view of the greater omentum in a 2.5-year-old Fleckvieh cow with peritonitis attributable to type-4 abomasal ulcer. The greater omentum is covered with ingesta and fibrin

All cows underwent portmortem examination and all had a type-4 abomasal ulcer and generalised peritonitis (Figs. 10, 11 and 12). Thirty-six cows also had a type-1 ulcer, six cows had a type-2 ulcer and one cow had a type-3 ulcer.

## Discussion

Forty-nine percent of all cases of type-4 abomasal ulcer occurred during the first four weeks of lactation, which had the highest incidence of all lactational stages. This was in general agreement with other reports [1, 6, 19–21]. Stressors affecting cows in the periparturient period, in addition to parturition, include the movement from the dry cow pen to the milking herd [22, 23] and the transition to a carbohydrate-rich ration [24], onset of lactation and a variety of typical early-lactation disorders [25]. These stressors are responsible for enhanced secretion of cortisol, hydrochloric acid and pepsin and also result in reduced secretion of prostaglandin E [25, 26]. Comorbidity was common in the present study and one third of all cows had one or more additional disorders. In an earlier study, 39 of 43 cows with perforated abomasal ulcer had one or more additional disorders [6]. Advanced pregnancy and early lactation in dairy cows is associated with increased stress, which was evidenced by increased hair cortisol concentrations in periparturient cows [27, 28] and in cows three weeks after parturition [27] compared with cows at other reproductive stages. Some of the cows had received NSAIDs and/or corticosteroids before referral; however, we were not able to determine when these drugs were given or the dosages used. It cannot be ruled out that these treatments were involved in the pathogenesis of the ulcers or in the perforation of a pre-existing type-1 or − 2 ulcer. Anti-inflammatory drugs such as corticosteroids and salicylic acid have been implicated in the pathogenesis of abomasal ulcer [7] and their use should be limited to 3 days. The clinical signs in cows with type-4 abomasal ulcer are associated with generalised peritonitis and have been described in many text books [7, 29–31], although details regarding the frequency of signs are sparse. The present study examined the clinical signs associated with a perforated ulcer in detail and identified compromised health status and anorexia (100%), abdominal guarding (81%), congested scleral vessels (77%), ruminal atony (73%), tachycardia (68%), tachypnoea (65%), positive foreign body tests (58%), decreased skin surface temperature (53%) and fever (49%) as the predominant clinical signs. However, with the exception of abdominal guarding and positive foreign body tests, these clinical signs may occur with many other disorders. Abdominal guarding and positive foreign body tests are pathognomonic for peritonitis, which is most often caused by traumatic reticuloperitonitis in cattle [32]. However, the same or similar signs of abdominal pain may also occur in disorders of the liver, omasum, lungs or, as shown here, the abomasum [32]. In the present study, 58% of the cows had one or more positive foreign body tests; the pole test was positive in 46% and the back grip in 40%. Of note, these frequencies were similar to those observed in 503 cows with traumatic reticuloperitonitis, in which the pole test was positive in 43% and the back grip in 39% of cows [14]. Taken together, these findings show that foreign body tests are not suitable for the differentiation of type-4 ulcer and traumatic reticuloperitonitis. Reduced or absent negative abdominal pressure detected during transrectal examination is an important clinical finding that suggests peritonitis [29] and occurred in 39% of the cows in the present study. Intraabdominal manoeuvrability with the examining arm is conspicuously increased compared with healthy cows [33]. Thus, type-4 abomasal ulcer is the most likely diagnosis when typical signs of peritonitis such as abdominal guarding, positive foreign body tests and fever are accompanied by a severely compromised health status, tachycardia, tachypnoea and other signs of shock. Of 503 cows with traumatic reticuloperitonitis, only 26% had tachycardia and 8% had tachypnoea [14] compared with 68 and 65% of the cows with type-4 ulcer. Tachycardia in cows with type-4 ulcer is attributable to shock and tachypnoea indicates pain.

Haematocrit and plasma protein are important laboratory variables in cows with type-4 ulcer. Increased haematocrit in 69% of cows indicated shock-induced haemoconcentration, but interestingly, this was accompanied by hyperproteinaemia in only 11% of cases. Usually an increase in haematocrit goes hand in hand with an increase in plasma protein concentration, but in the present study, 60% of cows had normal plasma protein concentrations and in 29% the concentration was even lower than normal. An increased haematocrit accompanied by normal or decreased plasma protein concentration is indicative of loss or active secretion of protein-rich fluid into the peritoneal cavity [34]. It constitutes an important diagnostic sign in cattle with clinical signs typical of peritonitis and reflects the enormous loss of fluid and protein into the peritoneal cavity as a result of generalised peritonitis associated with type-4 abomasal ulcer [6]. Hypoproteinaemia associated with haemoconcentration may also occur with protein-losing enteropathy. In contrast, an increased haematocrit was seen in only 12% and decreased protein concentration in only 1% of 503 cows with traumatic reticuloperitonitis [14]. Hyperfibrinogenaemia occurred in 45% of the cows in the present study. Fibrinogen is an acute-phase protein that may increase within two to three days of onset of an inflammatory disease [35]. Interestingly the glutaraldehyde coagulation test time was increased significantly (16 to 60 min) in 9% of the cows. In these cows, the secretion of protein-rich fluid into the peritoneal cavity led to inadequate concentrations of inflammatory proteins in blood and thus delayed coagulation. A lack of or a severe delay in coagulation with the glutaraldehyde test in cows with clinical signs typical of peritonitis should alert the clinician to hypoproteinaemia and to the likely diagnosis of generalised peritonitis associated with effusion into the peritoneal cavity. Similar to other infectious bovine diseases, the white blood cell count was difficult to interpret in cows with type-4 ulcer. Only 19% of the cows had leukocytosis and 35% had leukopenia. By comparison, in cows with traumatic reticuloperitonitis, 42% had leukocytosis and only 4% had leukopenia [14]. Leukocyte numbers vary with species and depend on the relationship between leukopoiesis and peripheral consumption, and can vary from severely decreased to severely increased in animals with an infectious disease [36]. Leukopenia in cows with type-4 ulcer is attributable to consumption of leukocytes that exceeds the compensatory capacity of the bone marrow, which is less efficient in cattle than in other species [37]. Massive consumption of leukocytes was also seen in cattle with toxic mastitis [38]. Stress-induced secretion of corticosteroids in cows with type-4 ulcer may have contributed to leukopenia. The metabolic acidosis that was diagnosed in 49% of the cows with type-4 ulcer was likely the result of severe dehydration and anaerobic metabolism associated with shock [1]. The determination of l-lactate is useful for the characterisation of shock but unfortunately this was not done. Likewise, azotaemia in 56% of the cows was caused by shock and thus was prerenal. Aciduria was likely due to catabolic metabolism of protein as a result of chronic anorexia.

All but one of 47 peritoneal fluid samples had abnormalities typical of peritonitis including increased specific gravity and/or increased protein concentration, green discolouration suggesting contamination with ingesta or a putrid odour. However, it should be remembered that the standard classification of transudate and exudate do not always apply in sick cattle [39–42]. In contrast, the glucose concentration of peritoneal fluid has been shown to be a very sensitive and specific diagnostic parameter for septic peritonitis. The glucose concentrations of blood and peritoneal fluid are usually similar but because of glucose metabolism by microbes, the concentration is lower in peritoneal fluid than in blood in cows with septic peritonitis [43]. D-dimer concentration is considered the best criterion for the diagnosis of peritonitis [43] and is < 0.60 mg/l in healthy cattle [44]. This is a fibrin degradation product and plays an important role in the diagnosis of coagulation disorders. Peritonitis in cattle is associated with massive synthesis of fibrin immediately accompanied by fibrinolysis, which results in an increase in d-dimer concentration. It has a diagnostic sensitivity of 96% and a specificity of 98% [43]. This implies that the determination of glucose and d-dimer concentrations in peritoneal fluid should be part of the diagnostic workup in a cow suspected of suffering from peritonitis. D-dimer determination was not part of the standard examination protocol used in the present study because most cases occurred before the relationship between peritoneal d-dimer concentration and peritonitis in cattle was described [43].

Ultrasonography was useful for visualising and assessing generalised peritonitis in 47 cows. The proximity of the abomasum to the reticulum showed reticular lesions in many cows with careful scanning of both sides. This underlines the importance of a thorough examination of the entire abdomen even in cases where lesions that support the tentative diagnosis are readily detected. For instance, when ultrasonographic examination is limited to the reticulum in cows with a history or clinical signs typical of traumatic reticuloperitonitis, the examiner runs the risk of missing changes associated with generalised peritonitis. Widespread peritonitic changes were seen in 26% of 503 cows with traumatic reticuloperitonitis [45]. Fibrinous changes of the abomasal serosa, which provide direct evidence of a type-4 ulcer, were seen in only seven cows; however, more extensive inflammatory lesions in the abomasal region also strongly suggest a perforated ulcer. Type-4 abomasal ulcers have not been visualised ultrasonographically, presumably because the ulcer itself is obscured by inflammatory lesions soon after perforation. On the other hand, the resolution of 3.5 to 5.0 MHz transducers that are routinely used may be insufficient to visualise small ulcers, and higher frequencies are associated with insufficient penetration depth of sound waves.

Cows with diffuse peritonitis attributable to a perforated abomasal ulcer respond poorly to treatment [4] and reports of successful treatment are scarce [46]. An ulcer can be resected but peritonitis is usually advanced and resistant to therapy [6]. All cows of the present study were euthanased or died peracutely; treatment was discontinued once diffuse peritonitis was confirmed.

## Conclusions

The diagnosis of type-4 abomasal ulcer based on clinical signs alone is difficult and therefore requires additional diagnostic procedures including the determination of the haematocrit and plasma protein concentration, abdominal ultrasonography and analysis of peritoneal fluid. In most cases, these steps lead to a correct diagnosis and allow timely euthanasia of the cow to prevent further suffering and unnecessary treatment costs. The dilemma faced by clinicians is that even though a correct diagnosis is usually possible, effective treatment of type-4 abomasal ulcer is not feasible. Efforts should therefore be made to minimise or eliminate ulcerogenic factors in cattle in later pregnancy and early lactation.

## Abbreviations

EDTA: Ethylenediamintetraacetic acid
Fig.: min: Minute
pH: pH value
sd: Standard deviation
